# Supplementary material for: Targeting Angiogenesis by Blocking the ATM–SerRS–VEGFA Pathway for UV-Induced Skin Photodamage and Melanoma Growth
Source: Cancers (Basel). 2019 Nov 22;11(12):1847. doi: 10.3390/cancers11121847 (PMC6966470; doi:10.3390/cancers11121847)
Supplement: Supplementary file 1 [file cancers-11-01847-s001.zip › cancers-631753-supplementary-final.docx]

Article

Targeting Angiogenesis by Blocking the
ATM–SerRS–VEGFA Pathway for UV-Induced
Skin Photodamage and Melanoma Growth

Yadong Song, Hongyan Lu, Qiong Wang and Rong Xiang

Supplementary Materials


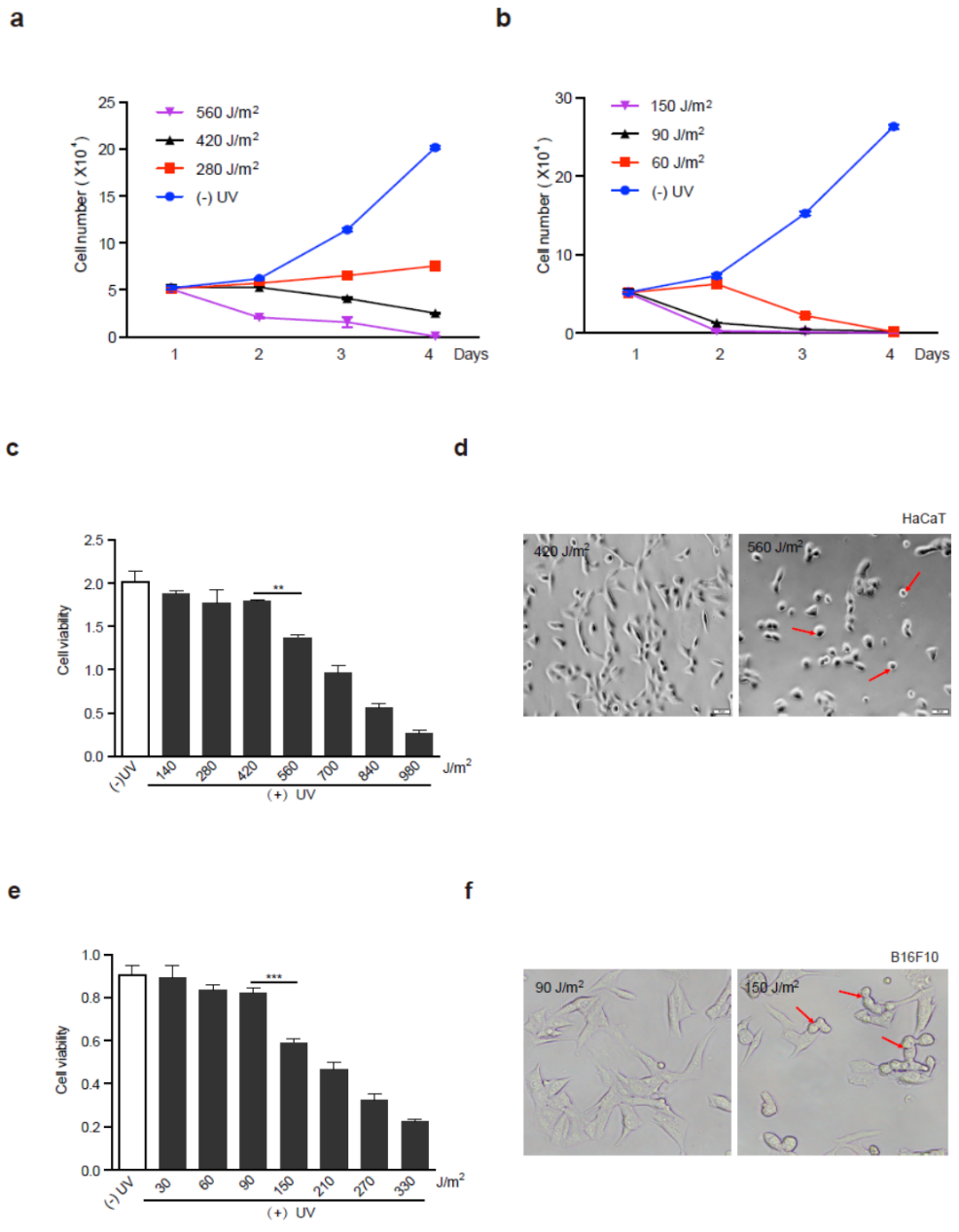


**Figure S1.** The growth and viability were inhibited by the high dose of UV radiation**.** (**a**) HaCaT cells were irradiated with indicated doses of UV and counted everyday. (**b**) B16F10 cells were irradiated with indicated doses of UV and counted everyday. (**c**) The viability of HaCaT cells was determined via CCK8 assay. (**d**) Representative images of HaCaT cells treated with different doses of UV. Red arrow: dying cells. Bar = 50 μm. (**e**) The viability of B16F10 cells was determined via CCK8 assay. (**f**) Representative images of B16F10 cells treated with different doses of UV. Red arrow: dying cells. Bar = 50 μm. All data above are presented as means ± SEM (*n* = 3, * *p* < 0.05, ** *p* < 0.01, *** *p* < 0.001) of three independent repeats.


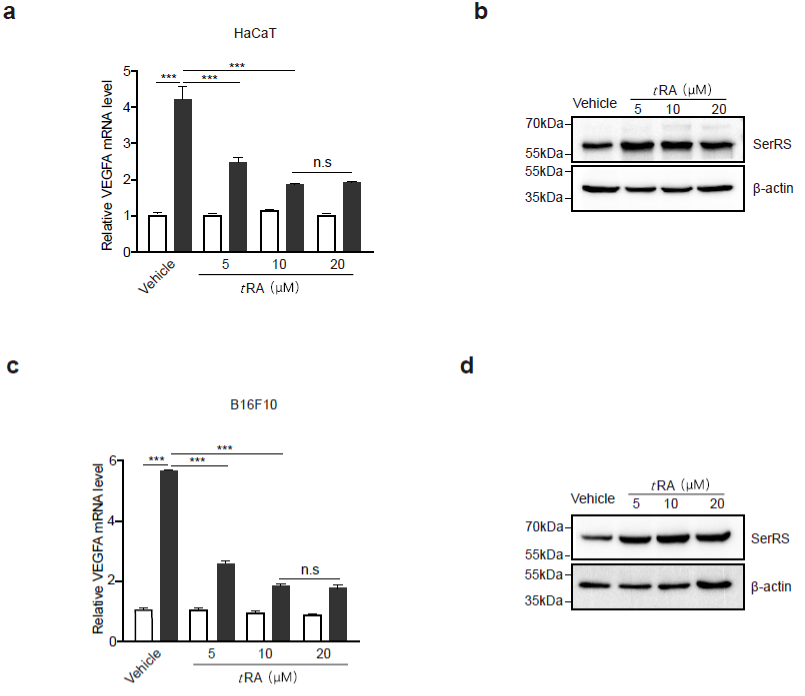


| Figure 2b. SerRS | Figure 2b. β-actin |
| --- | --- |
| **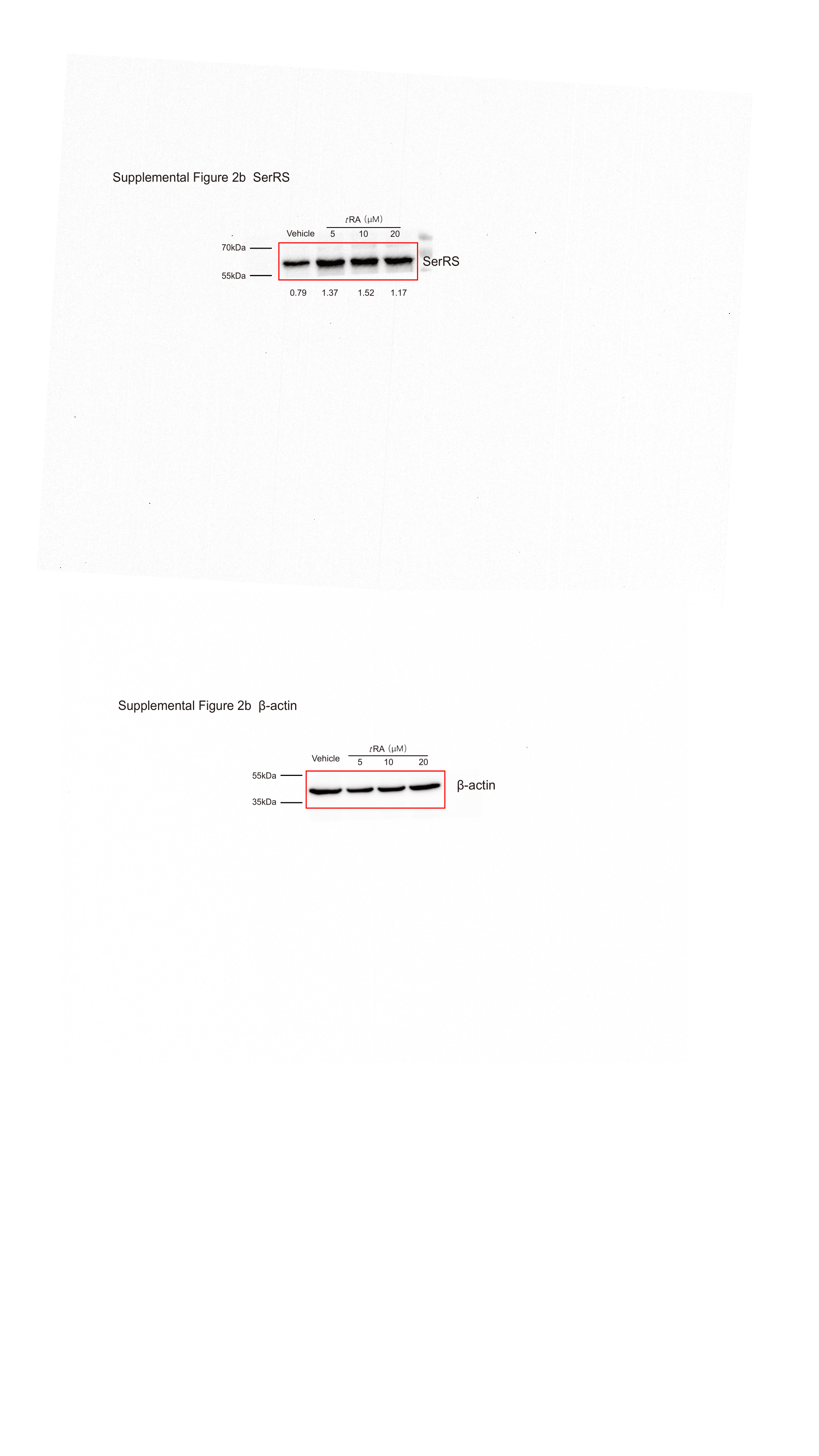** | 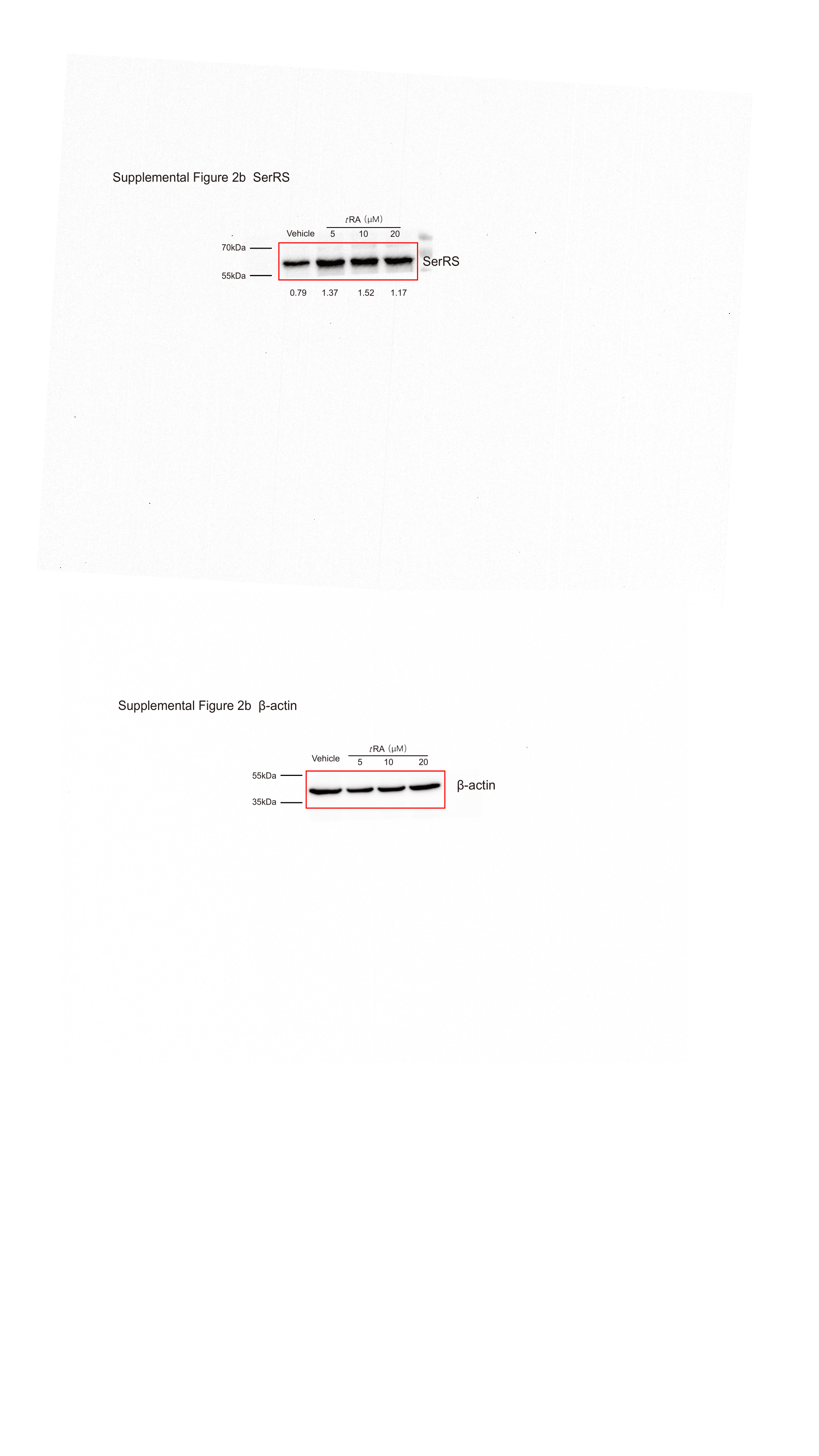 |
| Figure 2d. SerRS | Figure 2d. β-actin |
| 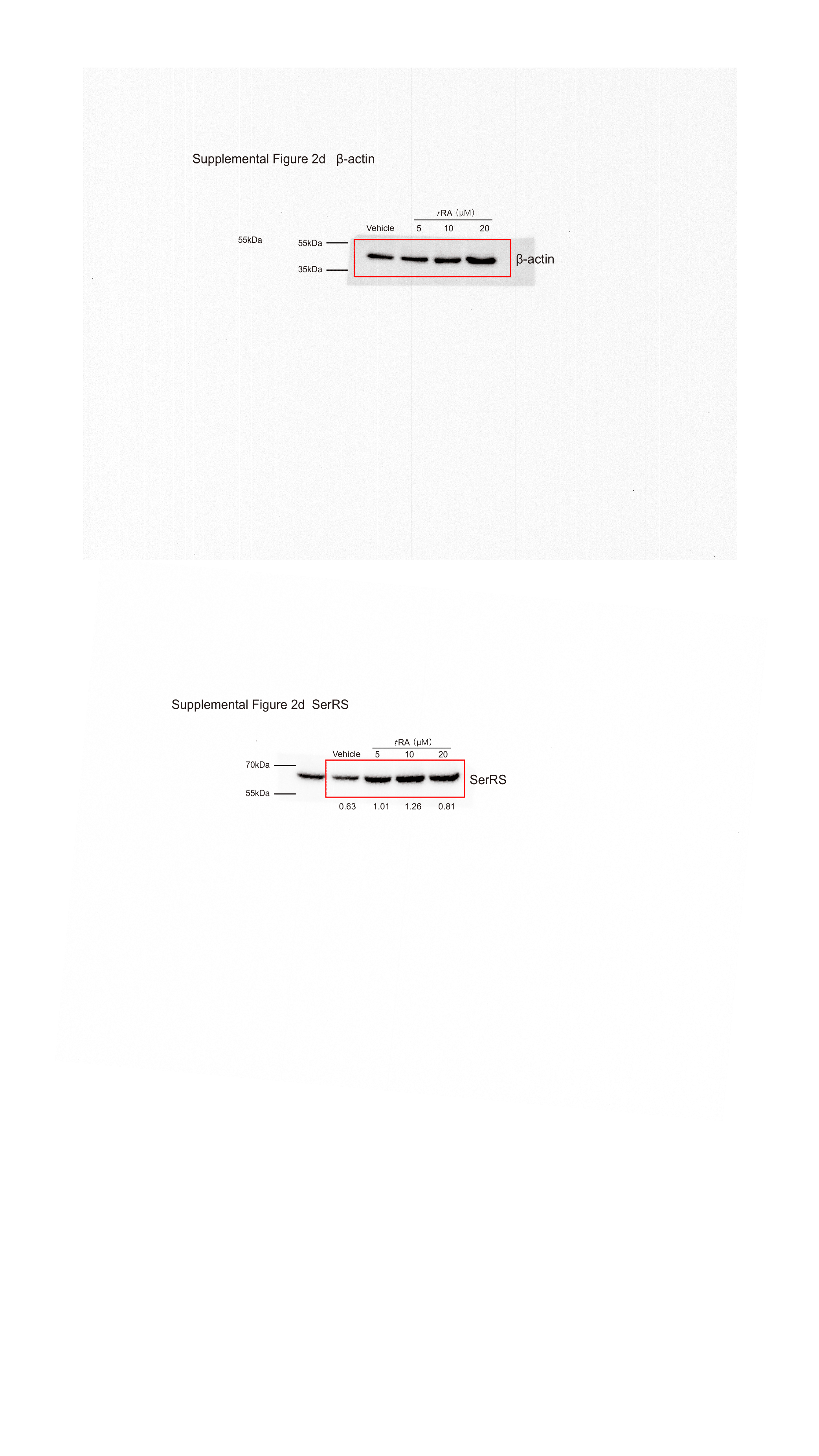 | 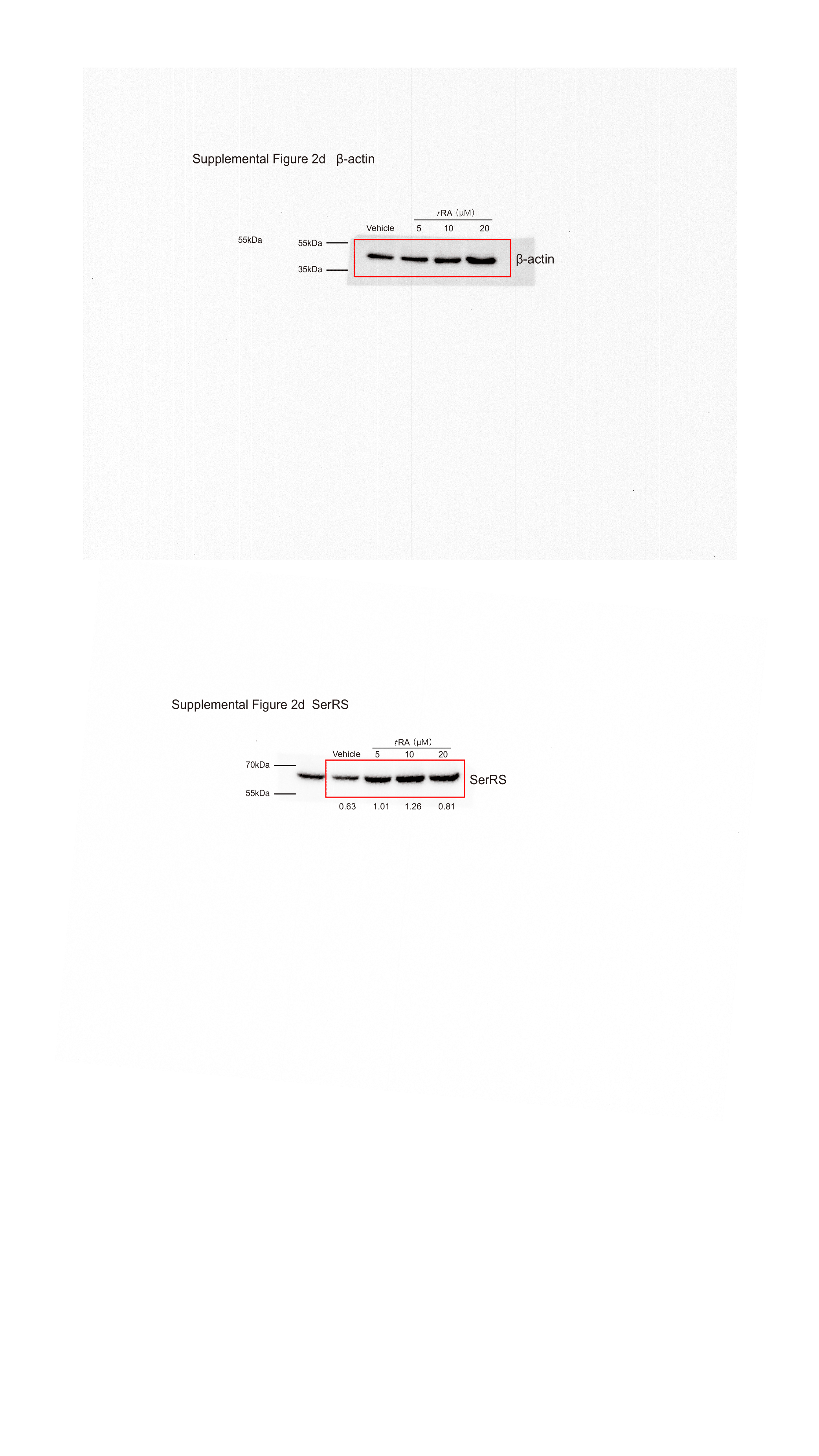 |

**Figure S2.** A higher dose of *t*RA didn’t yield either stronger SerRS induction or VEGFA expression**.** (**a**) mRNA levels of VEGFA in HaCaT cells were analyzed by RT-PCR. (**b**) Western blot analysis of SerRS in HaCaT cells, β-actin serves as a control. (**c**) mRNA levels of VEGFA in B16F10 cells were analyzed by RT-PCR. (**d**) Western blot analysis of SerRS in B16F10 cells, β-actin serves as a control. All data above are presented as means ± SEM (*n* = 3, * *p* < 0.05, ** *p*< 0.01, *** *p* < 0.001) of three independent repeats.


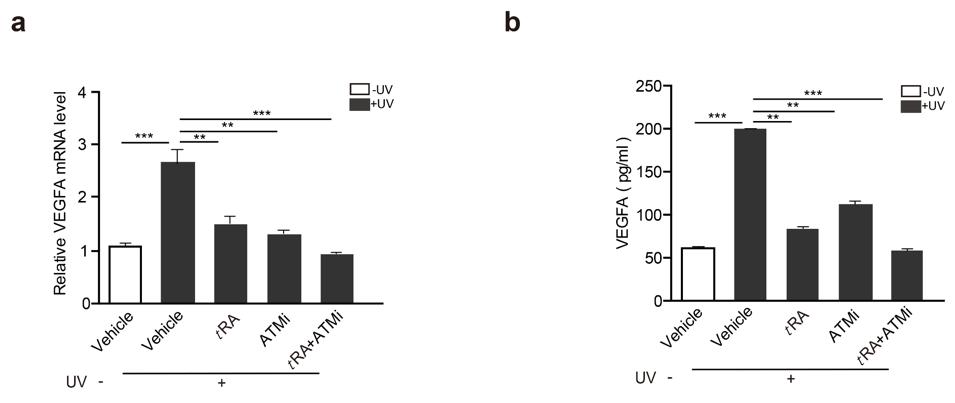


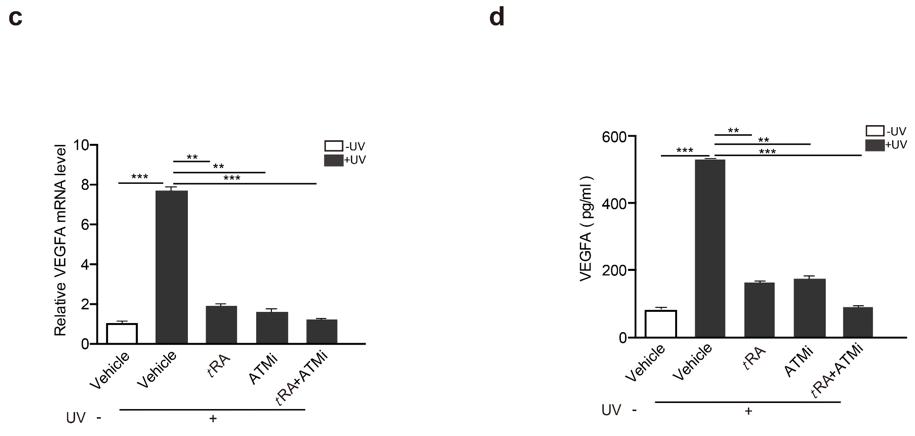


**Figure 3.** ATM inhibitor enhances the effect of *t*RA on the inhibition of UV-induced VEGFA expression in cells**.** (**a**) mRNA levels of VEGFA in HaCaT cells were analyzed by RT-PCR. (**b**) VEGFA secretion by HaCaT cells was determined via ELISA. (**c**) mRNA levels of VEGFA in B16F10 cells were analyzed by RT-PCR. (**d**) VEGFA secretion by B16F10 cells was determined via ELISA. All data above are presented as means ± SEM (*n* = 3, * *p* < 0.05, ** *p* < 0.01, *** *p* < 0.001) of three independent repeats.
