# Supplementary material for: Targeting Angiogenesis by Blocking the ATM–SerRS–VEGFA Pathway for UV-Induced Skin Photodamage and Melanoma Growth
Source: Cancers (Basel). 2019 Nov 22;11(12):1847. doi: 10.3390/cancers11121847 (PMC6966470; doi:10.3390/cancers11121847)

Figure 1d P-ATM

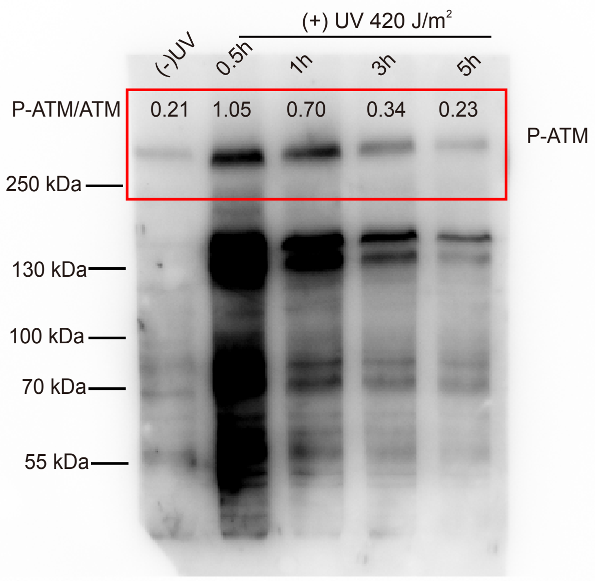

Figure 1d ATM

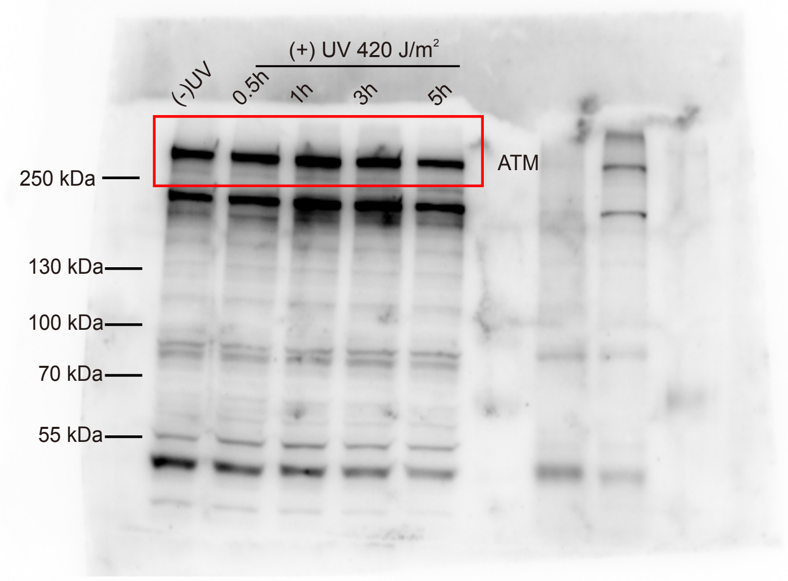

Figure 1d SerRS

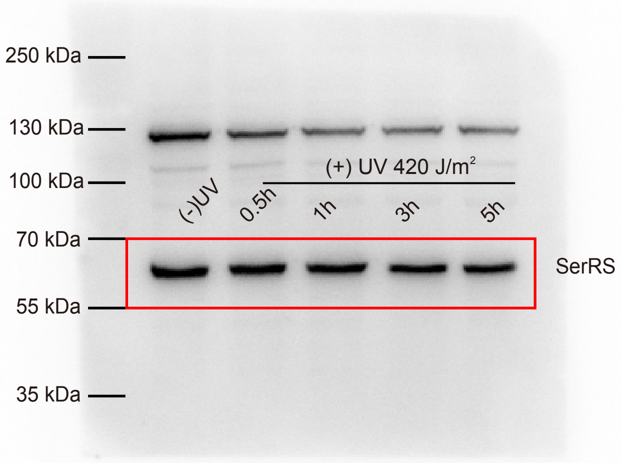

Figure 1d P-SerRS

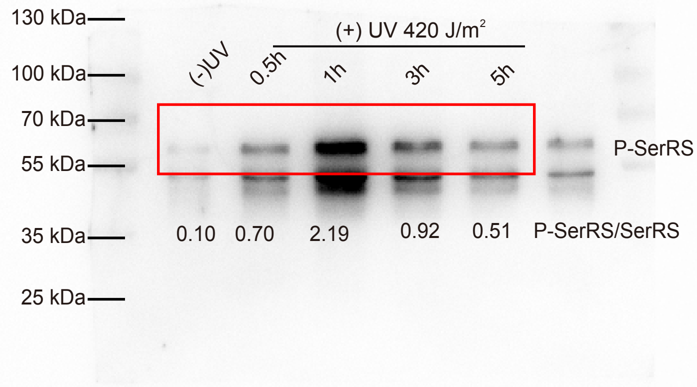

Figure 1d β-actin

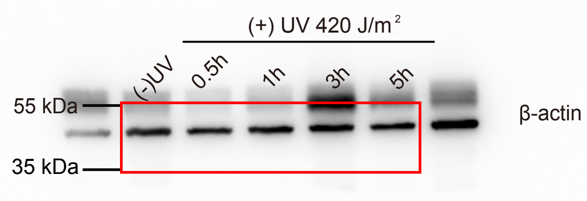

Figure 1h β-actin

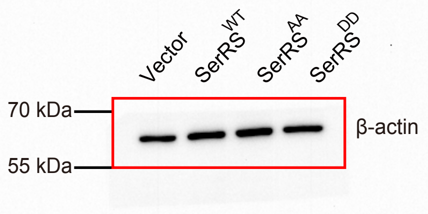

Figure 1h SerRS

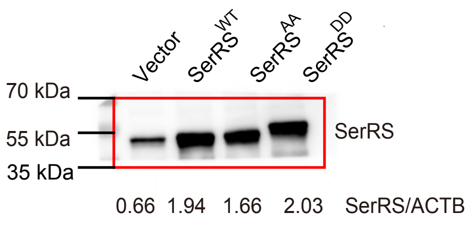

Figure 1e P-ATM

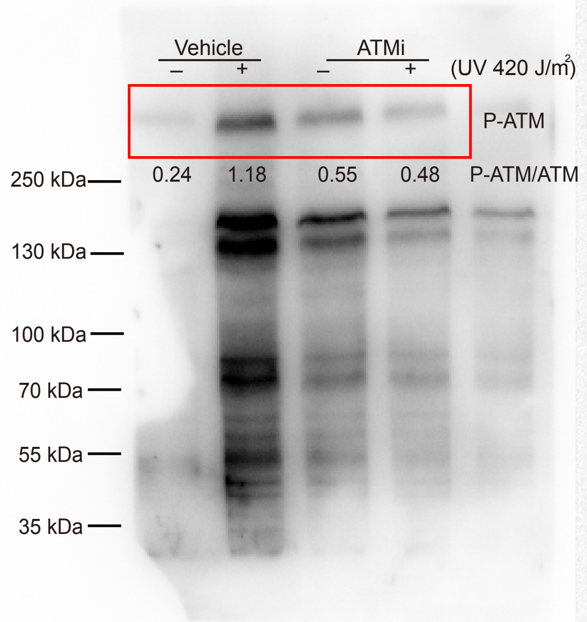

Figure 1e ATM

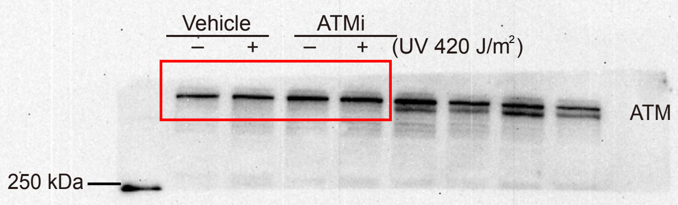

Figure 1e SerRS

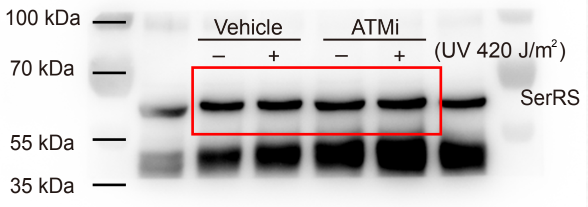

Figure 1e P-SerRS

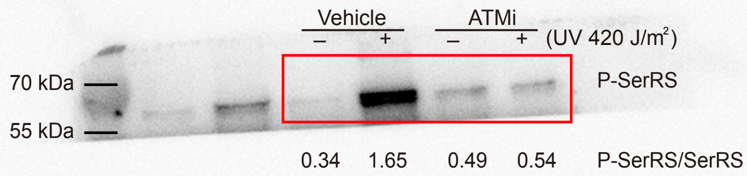

Figure 1e β-actin

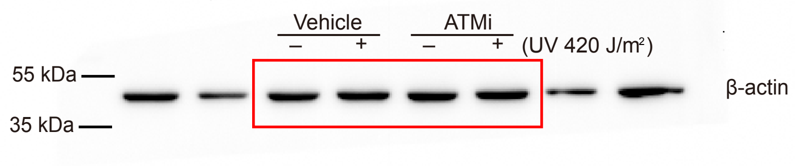

Figure 2g SerRS

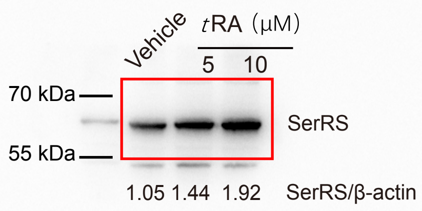

Figure 2g β-actin

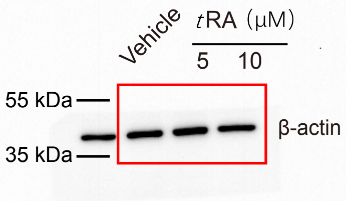

Figure 3c β-actin

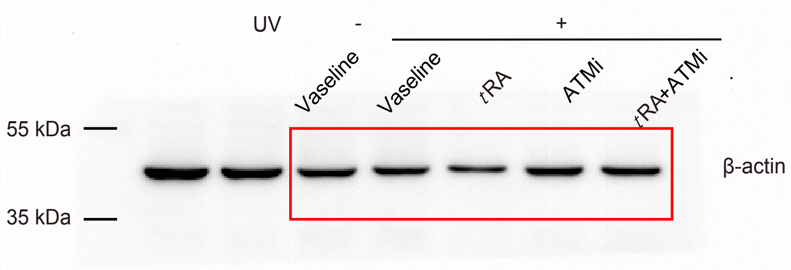

Figure 3c VEGFA

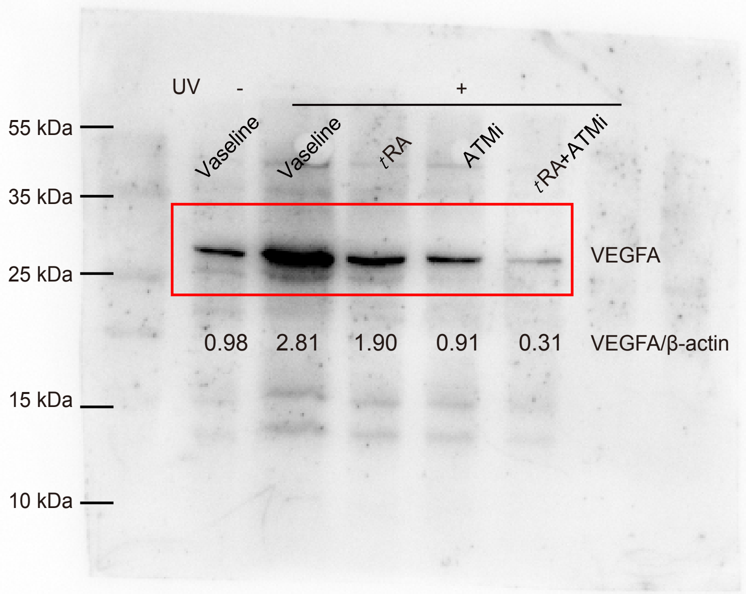

Figure 6c ATM

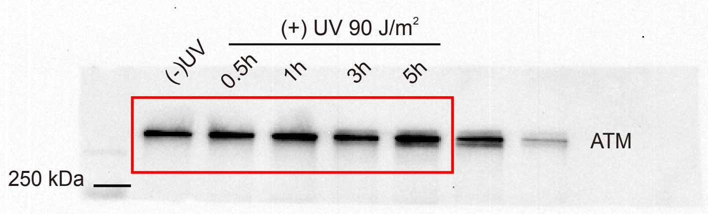

Figure 6c P-ATM

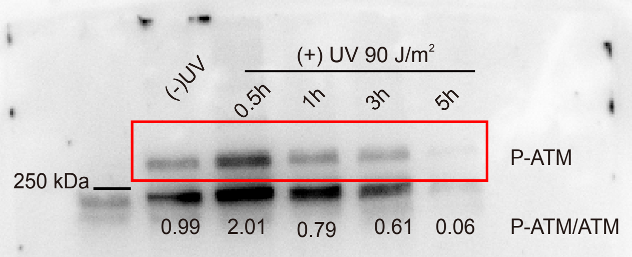

Figure 6c β-actin

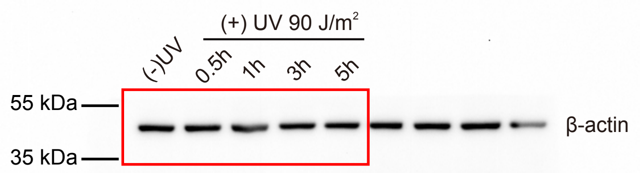

Figure 6c SerRS

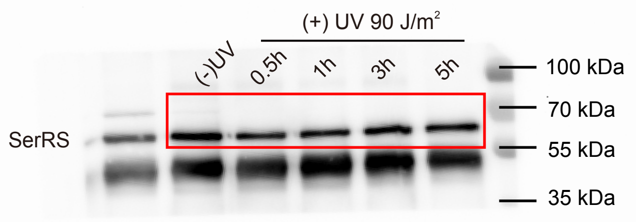

Figure 6c P-SerRS

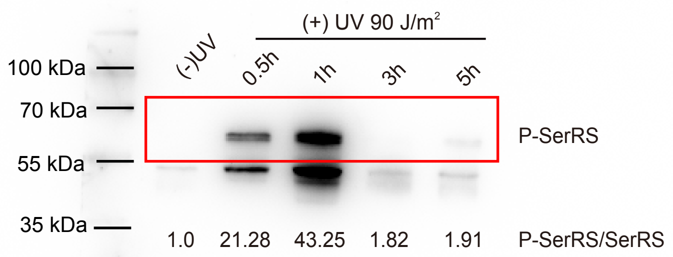

Figure 6d ATM

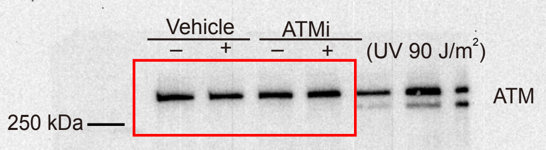

Figure 6d P-ATM

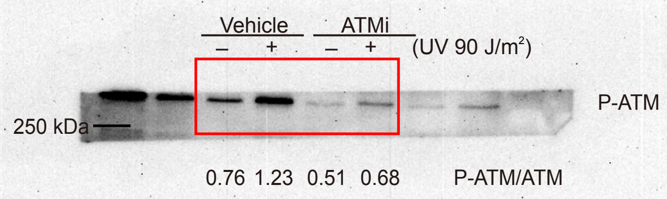

Figure 6d SerRS

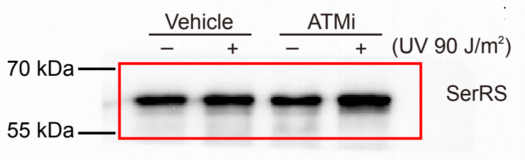

Figure 6d P-SerRS

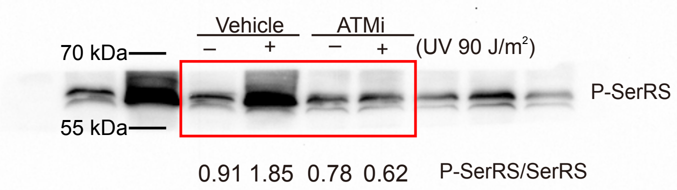

Figure 6g β-actin

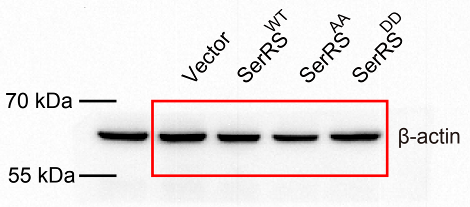

Figure 6g SerRS

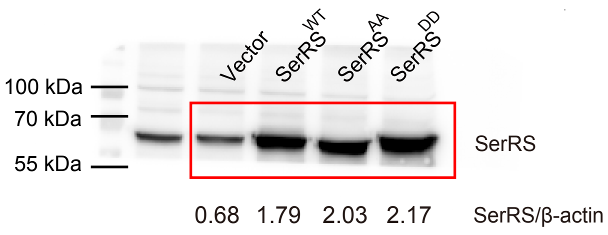

Figure 6j SerRS

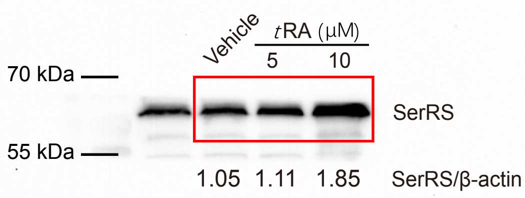

Figure 6j β-actin

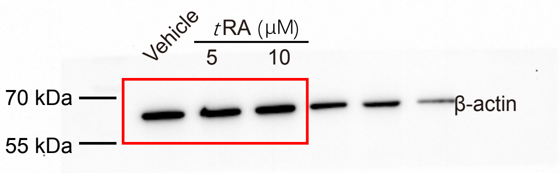

Figure 7e β-actin

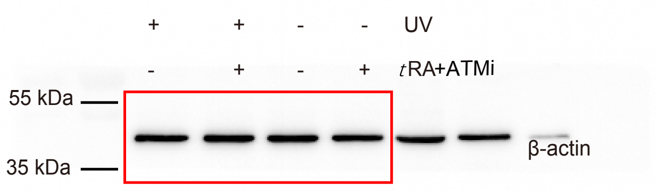

Figure 7e VEGFA

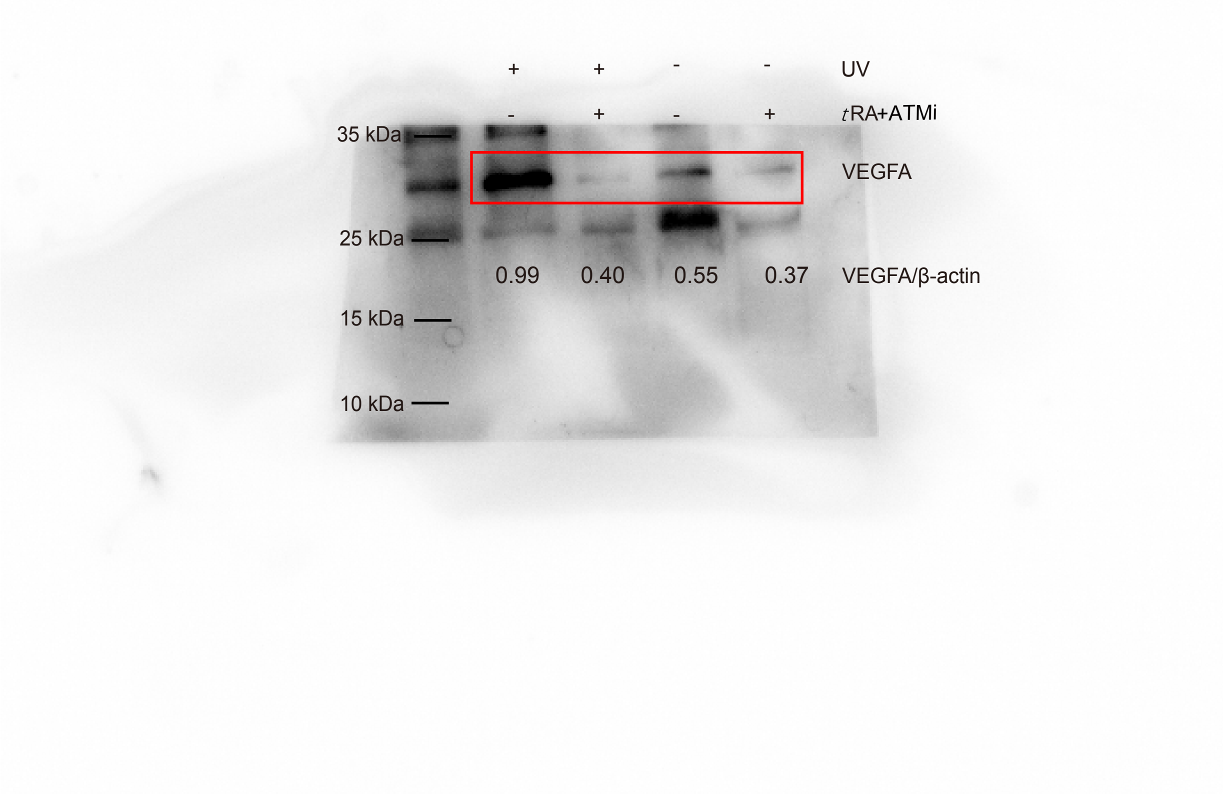

Supplement: Supplementary file 1 [file cancers-11-01847-s001.zip › cancers-631753-Western blot figures supplementary.pdf]
